# The Role of Perceived Sleep Quality in Mental and Cognitive Health among Older Residents in Subsidized Housing

**DOI:** 10.1093/geroni/igaf122.2592

**Published:** 2025-12-31

**Authors:** Yuri Jang, Juyoung Park, Seo-Yun Choi, Nan Sook Park, David A Chiriboga, Soondool Chung, Jung In Park, Sunmin Lee

**Affiliations:** University of Southern California, Los Angeles, California, United States; Emory University, Georgia, United States; University of Southern California, Los Angeles, California, United States; University of South Florida, Tampa, Florida, United States; University of South Florida, Tampa, Florida, United States; Ewha Womans University, Seoul, Republic of Korea; University of California, Irvine, Irvine, California, United States; University of California, Irvine, Irvine, California, United States

## Abstract

Given the importance of sleep health in later years and the greater likelihood of older immigrants with socioeconomic disadvantages experiencing sleep disturbances, we examined the impact of perceived sleep quality on mental and cognitive health among older Korean-American residents in subsidized housing. Data were collected from surveys with 318 older Korean Americans (Mean age = 79.5, SD = 6.66) living in subsidized housing in the Los Angeles area. Sleep quality was measured using a single-item self-rating (excellent/very good/good vs. fair/poor). Both screening tools and self-ratings were used for outcomes: mental health (measured by the Patient Health Questionnaire–9 [PHQ–9]) and cognitive health (measured by the Mini-Mental State Examination [MMSE] and self-rated cognitive health). Multivariate models assessed the mental and cognitive health risks associated with poor sleep quality. Poor sleep quality was a significant predictor in both models of probable depression, indicated by the PHQ–9, and self-rated mental health. However, the impact of sleep quality on cognitive health varied by measure: significance was found for self-rated cognitive health, but not for MMSE-based cognitive status. The discussion addresses the mental and cognitive health risks associated with poor sleep quality and potential reasons for the varied impact by measure. Findings suggest that efforts to promote health and well-being in senior housing should specifically address sleep. Improving sleep quality among senior housing residents may reduce health risks and enhance overall quality of life, empowering them to fulfill their desire for independent living and aging in place.

